# Experimental Study and Theoretical Analysis on the Compression–Shear Multiaxial Mechanical Properties of Recycled Concrete

**DOI:** 10.3390/ma15144810

**Published:** 2022-07-10

**Authors:** Yongping Zhang, Shuai Peng, Xiaoqing Du, Zhenpeng Yu, Jie Wu, Xinghua Xie, Yanli Hu

**Affiliations:** 1 School of Mechanics and Engineering Science, Shanghai University, Shanghai 200444, China; zhangyp01@shu.edu.cn (Y.Z.); dxq@shu.edu.cn (X.D.); yuzhenpeng@shu.edu.cn (Z.Y.); 2 Agriculture, Rural and Technology Bureau of Ping’an District, Haidong 810600, China; wujie_2021@163.com; 3 Nanjing Hydraulic Research Institute, Nanjing 210029, China; iamxiexh@163.com; 4 College of Civil Engineering, Yancheng Institute of Technology, Yancheng 224051, China; huyanli@ycit.edu.cn

**Keywords:** recycled concrete, experimental study, compression–shear multiaxial, stress mechanism, failure criterion

## Abstract

Recycled concrete, which is formed by replacing coarse aggregates in ordinary concrete with recycled aggregates (RA), is of great significance for the secondary utilization of waste building resources. In civil engineering, concrete structures are sometimes subjected to a compression–shear multiaxial stress state. Therefore, research on the compression–shear multiaxial mechanical properties of recycled concrete plays an important role in engineering practice. To explore the effect of RA replacement rate on the compression–shear properties of recycled concrete, an experimental study was carried out using a compression–shear testing machine and considering five RA replacement rates and five axial compression ratios. Consequently, the failure modes and mechanical property parameters under different working conditions were obtained and were used to analyze the effects of RA replacement rate and axial compression ratio on the shear stress of recycled concrete. Eventually, the following conclusions were reached: With the growth of axial compression ratio, the shear cracks exhibit a developing trend along the oblique direction, and the friction traces on the shear surface are gradually deepened. As the replacement rate increases, the number of shear cracks is gradually increased, accompanied by increasing broken fragments falling off from the shear interface. Since the action of the axial compression ratio can effectively improve the mechanical bite force and friction on the shear interface of recycled concrete, as the axial compression ratio increases, the shear stress is gradually increased. On the other hand, due to the initial damage of RA and its weak adhesion with cement mortar, the shear stress is gradually reduced with the increase of RA replacement rate. Meanwhile, the increase in shear stress shows a gradually decreasing trend with the growth of axial compression ratio. Specifically, for the RA replacement rates of 0% and 100%, the shear stress increased by 4.06 times and 3.21 times, respectively, under the influence of the axial compression ratio. Under different axial compression ratios, the shear stress was reduced by 43~46%, due to the increase of RA replacement rate. In addition, based on the octahedral stress space and the principal stress space, a compression–shear multiaxial failure criterion and shear stress calculation model for recycled concrete were proposed, by considering the effect of the RA replacement rate. The outcomes of this research are of great significance for engineering applications and the development of recycled concrete.

## 1. Introduction

Recycled concrete is a type of eco-friendly concrete made from demolished concrete structures, by replacing the coarse aggregates or fine aggregates in ordinary concrete with waste concrete materials after secondary treatment. By making full use of construction waste, the production of recycled concrete can effectively control environmental pollution and, therefore, has good application prospects, in view of its conformance to the needs of sustainable development [1,2]. During its service period, a concrete structure is often subjected to a complex stress state, which may have a great impact on the initiation and development of structural cracks. The compression–shear state is one of the common complex stress states for structures such as beams, columns, corbels, and tunnel linings [3,4]. Therefore, it is very important to comprehensively examine the mechanical properties of recycled concrete under compression–shear action [5].

For research on the mechanical properties of recycled concrete, Xuan. et al. [6] prepared a type of high-strength recycled concrete by utilizing waste masonry, and examined its mechanical properties, and the results indicated good application feasibility. Thomas, J. et al. [7] investigated the compressive and tensile mechanical properties of recycled concrete under different recycled aggregate (RA) replacement rates, and the results showed that the compressive strength and tensile strength of recycled concrete were slightly lower than that of ordinary concrete. In addition, the authors also examined the durability of recycled concrete and proposed a suitable range for the RA replacement rate, as well as a calculation model for determining the concrete mix ratio. Duan, Z. et al. [8] investigated the mechanism of influence of RA on the mechanical properties of concrete, from a micro-perspective, and analyzed the influence of replacement rate on the mechanical properties from a quantitative perspective. The results showed that the amount of RA added to the mortar was the key factor affecting the mechanical properties of recycled concrete. Silva, F.A. et al. [9] used recycled fine aggregates and recycled coarse aggregates to replace ordinary aggregates, considering different replacement rates, and found that the replacement of ordinary coarse aggregates with recycled coarse aggregates had a significant impact on the mechanical properties of concrete, but the replacement of ordinary fine aggregates with recycled fine aggregates had little effect on the overall performance. Lehner, P. and Horňáková, M. [10] examined the influence of fiber content and pre-compression stress on the chloride ion diffusion coefficient of recycled concrete structures. It was found that, when the fiber content exceeded the threshold level, the diffusion of chloride ions in recycled concrete structures became more obvious as the fiber content was increased and with the decrease of pre-compression stress. In view of the replacement of coarse aggregates with broken brick aggregates having a great effect on the mechanical properties of concrete, Rodsin, K. et al. [11] applied FRP to reinforce a recycled concrete column. The results indicated that, with the increase of FRP layers, the ultimate compressive stress and deformability of the recycled concrete column were significantly improved. On such basis, a corresponding calculation model for the ultimate compressive stress and deformation parameters was proposed. All the studies above examined the mechanical properties of recycled concrete through the perspective of uniaxial stress, but in practical engineering, concrete structures are often in a multiaxial stress state. Therefore, research on the uniaxial mechanical properties alone has obvious shortcomings, which may lead to the problem of insufficient or excessive use of material. Rukhaiyar, S. and Zeng, S. et al. [12,13] carried out a systematic study on the multiaxial mechanical properties of ordinary concrete and analyzed the influence of loading method and lateral stress on the multiaxial mechanical properties. On such a basis, a corresponding failure criterion and constitutive model were proposed. Yu, Z. et al. [14] conducted experimental research on the compression–shear multiaxial mechanical properties of ordinary concrete, and proposed a model to describe the relationship between the axial compression ratio and shear stress. Furthermore, a corresponding failure criterion was established based on the biaxial strength theory. Compared with the research on ordinary concrete, there are less literature reports on the multiaxial mechanical properties of recycled concrete. Yu, Z. et al. [15] examined the biaxial compression–compression and triaxial compression properties of recycled concrete using a true triaxial machine. By analyzing the influence of RA replacement rate and lateral stress on the mechanical properties, a corresponding failure criterion was proposed and the stress mechanism was revealed. Wang, Y. et al. [16] investigated the compression–shear multiaxial mechanical properties of recycled concrete by considering three replacement rates and established a corresponding constitutive model based on the test results. However, to date, the research on the multiaxial properties of recycled concrete still has obvious deficiencies, as the influence of replacement rate and axial compression ratio have not been fully elucidated, especially for low axial compression ratio conditions (which are more common in actual engineering applications). Therefore, it is necessary to conduct further research on the compression–shear multiaxial mechanics of recycled concrete.

In this paper, the compression–shear multiaxial properties of recycled concrete were examined using a hydraulic servo machine with combined compression and shear, and by considering five replacement rates and five axial compression ratios. The failure modes and mechanical property values were extracted from the test results and were used to analyze the influence of replacement rate and axial compression ratio on the compression–shear multiaxial mechanical properties of recycled concrete. Then, the corresponding failure mechanism was considered in detail. Furthermore, based on the octahedral stress space and the principal stress space, a compression–shear multiaxial failure criterion and a shear stress calculation model for recycled concrete were proposed. The study results are of great significance for engineering applications and the development of recycled concrete.

## 2. Materials and Methods

### 2.1. Specimen Preparation

The raw materials used in the recycled concrete mixture included water, cement, coarse aggregates and fine aggregates. Specifically, the water was urban natural water; the cement was ordinary Portland cement (P.O 42.5); the fine aggregates were river sand, with an average particle size of 0.3–0.5 mm, fineness modulus of 2.5, and apparent density of 2650 kg/m^3^; the natural aggregate crushed stone had a particle size of 4–16 mm, bulk density of 1470 kg/m^3^, apparent density of 2720 kg/m^3^, and mortar content of 0.00%; the recycled aggregates had a density of 1350 kg/m^3^, apparent density of 2560 kg/m^3^, and mortar content of 15.25%. In the preparation process, the coarse aggregates were treated by saturated water absorption first, and then all the specimens were poured and cured according to the specified requirements. The experiment was performed immediately after curing [17].

For the purpose of comprehensively examining the compression–shear multiaxial mechanical properties of recycled concrete, 5 RA replacement rates were selected in this paper, namely, 0% (i.e., ordinary concrete with a designed strength of C30), 25%, 50%, 75%, and 100%, by referencing to the replacement rates considered in the references [15,16]. According to the “Specification for mix proportion design of ordinary concrete (JGJ55-2011)”, the mixture ratio for C30 ordinary concrete was determined. The mix ratios of recycled concrete are shown in Table 1.

### 2.2. Loading Plan

The compression–shear experiment in this paper mainly aimed to investigate the influence of RA replacement rate and axial compression ratio on the shear mechanical properties of recycled concrete. The axial compression ratios to be tested were determined according to the uniaxial compressive strength ratio of recycled concrete under 5 different replacement rates, which were 0, 0.138, 0.207, 0.276, and 0.414. For the compression–shear loading process, the axial load was applied first, at the rate of 0.05 MPa/s, with the load-controlled method. After reaching the preset axial load value, the shear load began to be applied using the displacement-controlled method. The shear loading strain rate followed the static loading condition, i.e., 10^−5^/s. The shear loading stopped immediately upon the failure of the test specimen. The collection of load and displacement data began with the application of the shear load. Considering the limitations of the shear box used in this study and the shape of specimens used in the relevant literature [16,18], all the test specimens for the compression–shear multiaxial experiment were designed as 100 mm × 100 mm × 100 mm cubes. Taking into account the discreteness and randomness characteristics of concrete materials, 3 specimens were used for each loading condition, so as to calculate the mean value for analysis.

**Figure 1:**
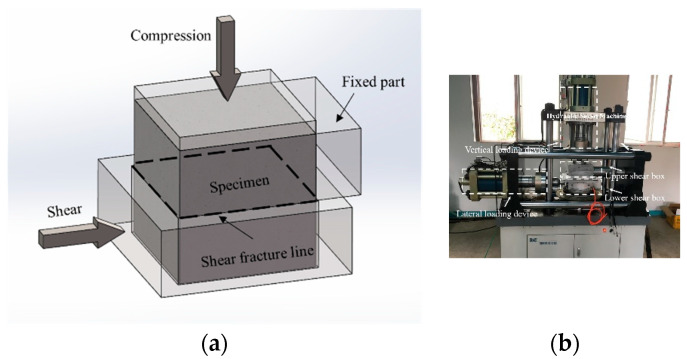
Loading schematics and loading devices. (**a**) loading device; (**b**) loading process.

In this study, the compression–shear loading was implemented using compression-shear hydraulic servo machines, which are mainly composed of a lateral loading hydraulic loading device and a vertical hydraulic loading device. The maximum displacements were both 100 mm, and the maximum load of the lateral loading device and the vertical loading device were both 100 tons. Each loading device was equipped with independent load sensors and displacement sensors, whose maximum errors met the test requirements. After placing the specimen in the shear box, the vertical hydraulic loading device exerted axial compression on the specimen, while the lateral loading device exerted a shearing action on the shear surface of the specimen. This loading method conformed to the requirements specified in the experiment plan. The loading schematics and loading devices are shown in Figure 1.

## 3. Analysis of Test Results

### 3.1. Failure Mode

**Figure 2:**
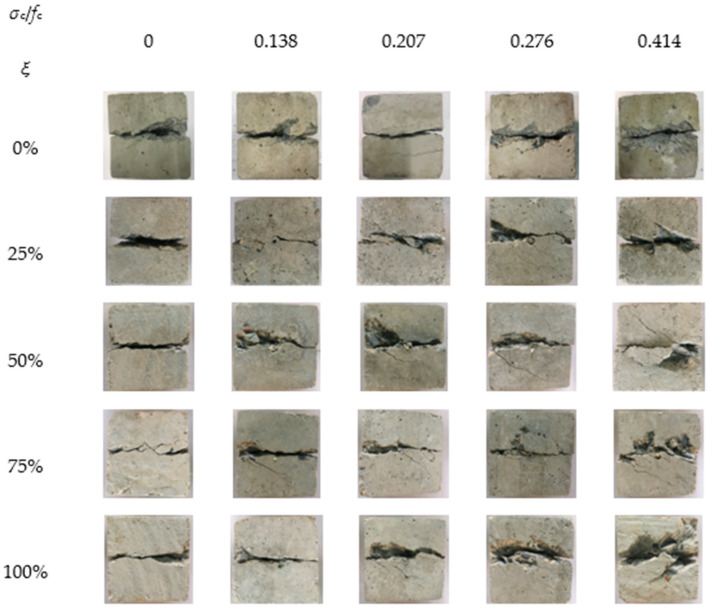
Shear crack failure modes of recycled concrete.

**Figure 3:**
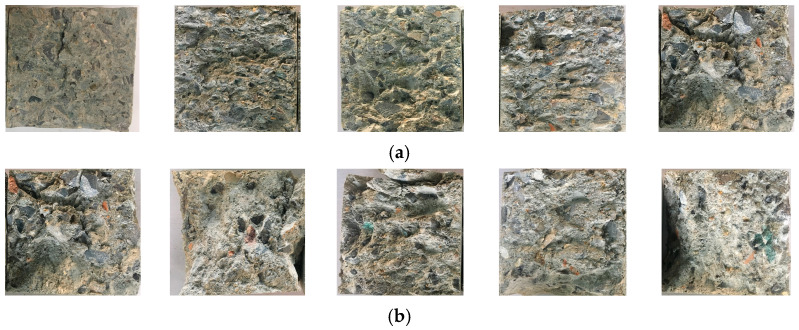
Shear interface failure modes of recycled concrete. (**a**) The influencing factor of recycled aggregate (RA) replacement rate: 0%, 25%, 50%, 75% and 100% (zero axial compression ratio); (**b**) The influencing factor of axial compression ratio: 0, 0.138, 0.207, 0.276 and 0.414 (100% RA replacement rate).

The shear failure modes of recycled concrete under different loading conditions were obtained from the experiment, which were used to study the influencing trends of the lateral compressive stress and RA replacement rate on the shear crack failure mode and shear interface failure mode of the recycled concrete, *σ_c_*/*f_c_* represents the axial compression ratio and *ξ* represents the replacement rate, as shown in Figure 2 and Figure 3.

As shown in Figure 2, when the axial compression ratio was zero, a roughly straight shear failure crack was formed under the shearing action. As the axial compression ratio increased, a certain number of fine cracks formed in the vicinity of the shear failure crack, which generally showed a developing trend along the oblique direction. When the axial compression ratio was high, the number and size of oblique cracks around the shear failure crack were both significantly increased. The mechanism for this phenomenon can be explained as follows: The axial load exerts a compressive force on the shear surface of the test specimen, so that the shear surface is subjected to the combined force of compression and shear. As the axial compression ratio increases, this combined force is gradually increased and will eventually result in the failure mode, as mentioned above. For specimens with different RA replacement rates, the shear failure crack basically exhibits similar variation trends under the influence of axial compression ratio. A slight difference lies in that, at a higher replacement rate, the number and size of oblique cracks are relatively larger and the specimen is more prone to generating broken fragments. When the axial compression ratio is zero, a roughly straight line parallel to the shearing direction tends to be formed under the dislocation between the upper and lower shear box. This is because the two shear boxes can have a great constraining effect on the test specimen. Under the shearing action, the dislocation relative to each other will lead to the generation of shear failure cracks on the contact interface between the two boxes. Since the axial compression ratio is zero, there is no compression acting on the specimen in the axial direction. Thus, a relatively straight shear failure crack will be generated under the shearing action. When the axial compression ratio is not zero, the specimen is prone to generating oblique cracks during the shear loading process, under the action of the axial compression ratio. In general, the larger the axial compression ratio, the more obvious the oblique cracks. Yu et al. [14] carried out experimental research on the compression–shear multiaxial mechanical properties of ordinary concrete by setting the highest axial compression ratio as 0.09. When the axial compression ratio was low, the shear crack failure modes under different conditions were basically similar. Wang et al. and Liu et al. [16,18] examined the compression–shear multiaxial mechanical properties of recycled concrete, by focusing on high axial compression ratio conditions. When the axial compression ratio was high, it was found that the failure modes were significantly different from those under low axial compression ratio conditions. This is consistent with the conclusions in this paper.

As it can be seen from Figure 3, with the growth of axial compression ratio, the friction traces on the failure interface were gradually deepened, accompanied by an increase of broken fragments falling off. This was mainly because the axial compression produces a frictional force on the shear interface during the shearing process, and a higher friction effect will result in deeper friction traces and more broken fragments. As the replacement rate increases, the number of broken fragments falling off from the failure interface is gradually increased and the failure mode appears to be more serious. The mechanism for this phenomenon can be explained from two perspectives: First, the surface of the RA is attached with a certain amount of mortar, which leads to a poor bonding performance between the mortar and RA. Second, the RA contain more initial defects than natural aggregate crushed stone. Yu et al., Wang et al., and Liu et al. [14,16,18] studied the compression–shear multiaxial mechanical properties of ordinary concrete, recycled concrete, and coral concrete. According to their results, the developing trend of the shear interface failure modes with the growth of axial compression ratio were consistent with the conclusions in this paper. It can be seen that the action of axial compression ratio can effectively increase the friction on the shear interface, which will eventually result in the above-mentioned failure modes.

### 3.2. Stress–Strain Curve

**Figure 4:**
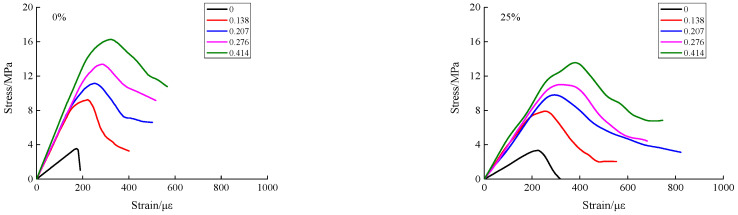

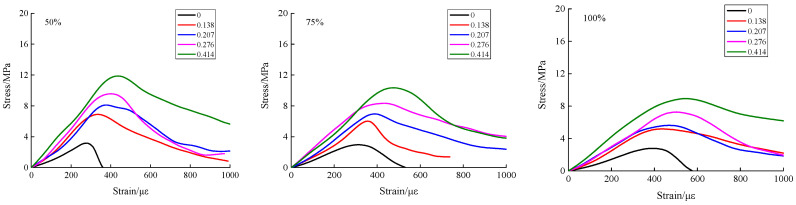
Shear stress–strain curves of recycled concrete with different replacement rates.

From the compression–shear experiment of recycled concrete in this paper, full shear stress–strain curves corresponding to the different axial compression ratios and RA replacement rates were obtained, as shown in Figure 4.

Figure 4 shows the shear stress–strain curves of recycled concrete with different RA replacement rates. From a preliminary analysis, it can be seen that there was no significant effect on the replacement rate of RA, but the axial compression ratio had an important effect on the developing trend of the shear stress–strain curve. When the axial compression ratio was zero, the descending section of the shear stress–strain curve developed rapidly, showing typical brittle failure characteristics. When the axial compression ratio was not zero, the descending section exhibited a gentle developing trend, showing obvious ductility characteristics. This was mainly because axial compression produces a friction effect on the shear surface, which delays the shearing failure process to a certain extent. According to the overall developing trend, the shear stress was gradually increased as the axial compression ratio grows. Moreover, with the increase of the RA replacement rate, the shear stress showed a gradually-decreasing trend, while the plastic deformability was gradually strengthened. Yu et al. and Liu et al. [14,18] carried out experimental research on the compression–shear multiaxial mechanical properties of ordinary concrete and coral concrete, and obtained shear stress–strain curves under different axial compression ratios. For all kinds of concrete, the influencing pattern of axial compression ratio on the developing trend of stress–strain curve was basically consistent with the conclusions in this paper.

### 3.3. Shear Stress

**Figure 5:**
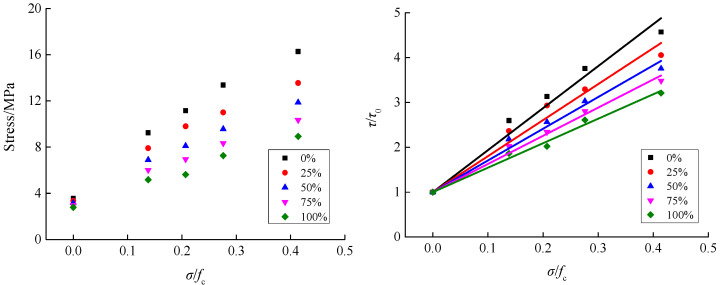
Variation trend of the influence of axial compression ratio on the shear stress of recycled concrete.

**Figure 6:**
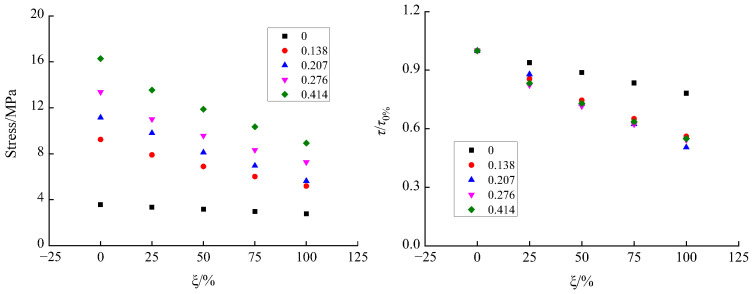
Variation trend of the influence of RA replacement rate on the shear stress of recycled concrete.

From the shear stress–strain curves of recycled concrete under different loading conditions, as shown in Figure 4, the peak stress points (i.e., the shear stress) were extracted, to analyze the variation trends of the influence of axial compression ratio and RA replacement rate on the shear stress of recycled concrete, as shown in Figure 5 and Figure 6.

Figure 5 illustrates the variation trend of the influence of axial compression ratio on the shear stress of recycled concrete. For a constant RA replacement rate, the shear stress gradually increased with the increasing axial compression ratio. When the replacement rate was 0%, the shear stress increased, from 3.56 MPa at the axial compression ratio of 0 to 16.27 MPa at the axial compression ratio of 0.414, indicating an increase of 4.57 times. When the replacement rate was 25%, 50%, 75%, and 100%, the shear stress of recycled concrete increased by 4.06 times, 3.76 times, 3.48 times, and 3.21 times, respectively, under the influence of the axial compression ratio. From the overall trend, it can be seen that the increase in shear stress due to the growth of axial compression ratio gradually reduced as the replacement rate increased. Under the action of axial compression, the mechanical bite force effectively improved, which further improved the shear stress of the recycled concrete. A higher axial compression corresponds to a higher mechanical bite effect, and consequently, the shear stress gradually increased under all replacement rates, with a growing axial compression. The main difference between RA and natural aggregate crushed stone is that the RA are attached with a certain amount of old mortar. Meanwhile, RA contains some initial defects, so its cylinder compressive strength is significantly lower than that of natural aggregate crushed stone. Thus, under the action of axial compression, recycled concrete is more prone to the generation of plastic damage. Therefore, the increase in shear stress of recycled concrete is significantly lower than that of ordinary concrete under the action of axial compression. Moreover, with an increase of RA replacement rate, the influence of axial compressive stress on the shear stress of recycled concrete is gradually weakened. Yu et al. [14] investigated the compression–shear multiaxial mechanical properties of ordinary concrete. When the axial compression ratio was 0.09, the shear stress was increased by 2.57 times compared with the case of no axial compression. Wang et al. [16] examined the range of shear–stress variation in recycled concrete under axial compression ratio, considering 0%, 50%, and 100% RA replacement rates. The results showed that the shear stress of recycled concrete was gradually increased with the growth of the axial compression ratio. When the axial compression ratio was high, with further growth of the axial compression ratio, the increase in shear stress slowed down. The above-mentioned research findings are basically consistent with the conclusions in this paper.

According to Figure 5, the shear stress of recycled concrete basically showed a linear relationship with the growth of the axial compression ratio for all five different RA replacement rates. When the axial compression ratio was zero, the shear stress was no longer affected by the axial compression ratio. Thus, the relationship between the axial compression ratio and the shear–stress variation factor can be expressed by Equation (1).

(1) $$\frac{\tau }{\tau_{0}}=1+a\times \frac{\sigma }{f_{c}}$$

where *τ* is the concrete shear stress and *τ*_0_ is the concrete shear stress when the axial compression ratio is 0; *σ* is the concrete axial stress; *f*_c_ is the concrete uniaxial compressive strength; and a is an undetermined coefficient.

By applying Equation (1) to perform mathematical regression analysis on the data of the axial compression ratio and shear stress of recycled concrete specimens with different RA replacement rates, the relationship between the axial compression ratio and shear–stress variation factor and the value of a were obtained, as shown in Figure 5 and Figure 6.

**Figure 7:**
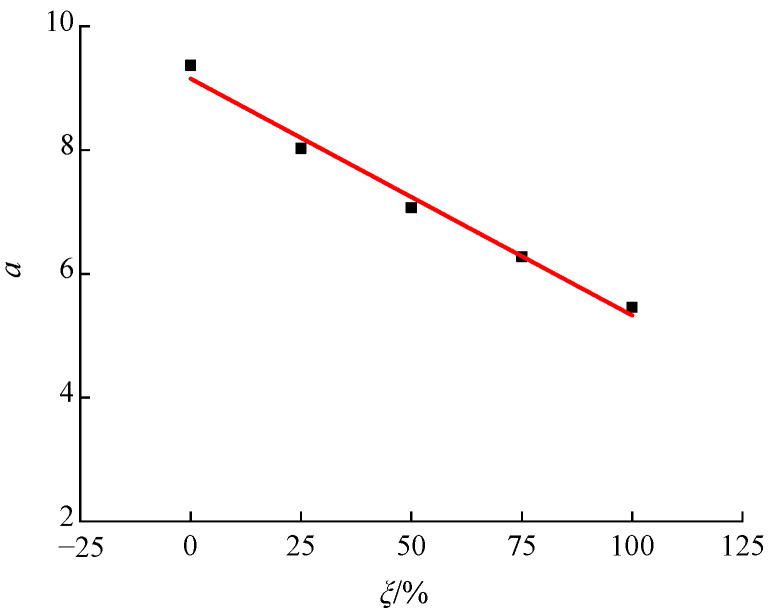
Relationship between the RA replacement rate and the parameter *a*.

According to Figure 5 and Figure 7, the proposed Equation (1) can well illustrate the relationship between the shear–stress variation factor and the axial compression ratio of recycled concrete with different RA replacement rates. From the qualitative analysis of the replacement rate *ξ* and the parameter a, the two showed a linear relationship. Then, mathematical regression analysis was carried out on the corresponding data, and the expression as shown in Figure 7 and Equation (2) was obtained.

(2) $$a=9.16-0.0383\times \xi ,\;R^{2}=0.9826$$

By substituting Equation (2) into Equation (1), the relationship between the shear–stress variation factor and the axial compression ratio of recycled concrete under the influence of RA replacement rate was obtained, as can be seen in Equation (3).

(3) $$\frac{\tau }{\tau_{0}}=1+(9.16-0.0383\times \xi )\times \frac{\sigma }{f_{c}}$$

Figure 6 shows the variation trend of the influence of axial compression ratio on the shear stress of the recycled concrete. For a constant axial compression ratio, the shear stress of the recycled concrete showed a gradually-decreasing trend with an increasing RA replacement rate. When the axial compression ratio was zero, the shear stress decreased from 3.56 MPa at the replacement rate of 0% to 2.78 MPa at the replacement rate of 100%, indicating a reduction by 21.83%. For the axial compression ratios of 0.138, 0.207, 0.276, and 0.414, the shear stress was reduced by 43.93%, 49.54%, 45.69%, and 45.11%, respectively, under the influence of the RA replacement rate. It can be seen from the overall trend that, when the axial compression ratio was zero, the reduction in shear stress due to the increase of RA replacement rate was generally larger than that under the condition when the axial compression ratio was not zero. For the various working conditions when the axial compression ratio was not zero, the reduction in shear stress due to the increase of replacement rate was basically similar. The mechanism can be explained as follows: Since the surface of the RA is attached with a layer of mortar, the bonding performance between the RA and the mortar is relatively weak; meanwhile, the RA contains certain initial defects, so its cylinder compressive strength is lower than that of the natural aggregate crushed stone. Consequently, the shear stress of recycled concrete shows a gradually-decreasing trend with an increasing RA replacement rate. Wang et al. [16] examined the influencing pattern of RA replacement rate on the shear stress of recycled concrete. It was reported that the shear stress of recycled concrete increased at first and then began to decrease as the replacement rate increased, and the same trend was observed under all axial compression ratios. There are some deviations between the conclusions in the references and the conclusions in this paper, which are mainly due to the fact that the physical properties of different RAs and different pretreatment methods have obvious effects on the mechanical properties of recycled concrete.

### 3.4. Shear Strain

**Figure 8:**
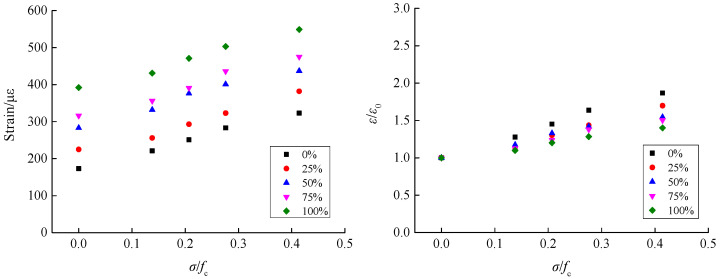
Variation trend of the influence of the axial compression ratio on the shear strain of recycled concrete.

**Figure 9:**
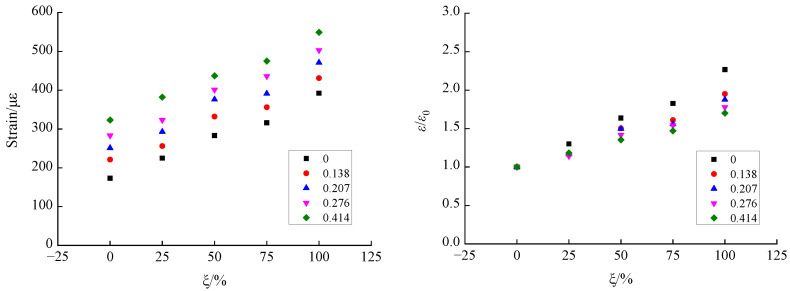
Variation trend of the influence of RA replacement rate on the shear strain of recycled concrete.

According to the shear stress–strain curves for recycled concrete at different axial compression ratios, based on different RA replacement rates, corresponding peak strain data for shear stress (i.e., the shear strain) was extracted to analyze the influence of the axial compression ratio and RA replacement rate on the shear strain of recycled concrete, as shown in Figure 8 and Figure 9.

Figure 8 illustrates the variation trend of the influence of axial compression ratio on the shear strain of recycled concrete. It can be seen that the shear strain of recycled concrete showed a gradually-increasing trend with a growing axial compression ratio, and this trend was observed under different RA replacement rates. When the replacement rate was 0%, the shear strain increased from 173 *με* at the axial compression ratio of 0 to 323 *με* at the axial compression ratio of 0.414, indicating an increase of 1.87 times. When the replacement rates were 25%, 50%, 75%, and 100%, the shear strain increased by 1.70 times, 1.54 times, 1.50 times, and 1.40 times, respectively, under the influence of the axial compression ratio. It can be seen from the overall trend, that the increase in shear strain of recycled concrete due to the growth of axial compression ratio showed a gradually-reducing trend with the increase of replacement rate. This is mainly because, under the action of the axial compression ratio, the plastic development ability of concrete is effectively increased, which suppresses the development and evolution of shear cracks and, consequently, improves its plastic deformability and ductility characteristics. Thus, the strain shows a gradually-increasing trend with a growing axial compression ratio. Due to the relatively weak interface between the RA and the mortar, as well as the initial defects in the RA, under the action of axial compression, the test specimen was prone to the formation of a certain degree of damage in the axial direction, which had an adverse effect on inhibiting the development of shear cracks. Correspondingly, the increase in plastic deformability was reduced, eventually leading to the phenomenon whereby the increase in shear strain of the recycled concrete under the influence of axial compression ratio was gradually reduced with the increase of the RA replacement rate. Yu et al. and Liu et al. [14,18] investigated the compression–shear multiaxial mechanical properties of ordinary concrete and coral concrete. The results showed that the shear stress was gradually increased with the growth of axial compression ratio, and the action of the axial compression ratio could effectively improve the plastic deformability of the concrete. This finding is consistent with the conclusion in this paper, regarding the effect of axial compression ratio on the shear strain of concrete.

Figure 9 illustrates the variation trend of the influence of the RA replacement rate on the shear strain of recycled concrete. The results show that the shear strain showed a gradually-increasing trend with an increasing replacement rate, and the same trend was observed under different axial compression ratios. When the axial compression ratio was zero, the shear stress increased from 173 *με* at the replacement rate of 0% to 392 *με* at the replacement rate of 100%, indicating an increase of 2.27 times. For the axial compression ratios of 0.138, 0.207, 0.276, and 0.414, the shear stress increased by 1.95 times, 1.88 times, 1.78 times, and 1.70 times, respectively, under the influence of the RA replacement rate. According to the overall trend, as the axial compression ratio increased, the increase in shear strain of the recycled concrete due to the increase of the RA replacement rate was gradually reduced. The underlying mechanism can be explained as follows: Due to a weak bonding performance with mortar, the deformability of RA is higher than that of natural aggregate crushed stone, which leads to the phenomenon whereby the shear strain of recycled concrete is gradually increased with an increasing replacement rate. In view of this, the growth of the axial compression ratio can effectively improve the mechanical bite force at the shear interface, and the negative effect caused by the weak bonding performance between the RA and the mortar can be counteracted to a certain extent. Eventually, the increase in shear strain of recycled concrete due to the increase of RA replacement rate is gradually reduced with a growing axial compression ratio. Wang et al. [16] studied the pattern of influence of RA replacement rate on the shear strain of recycled concrete. The results indicated that the shear strain of recycled concrete showed a gradually increasing trend with the increase of replacement rate, and the same trend was observed under all axial compression ratios. This finding is consistent with the conclusions in this paper.

## 4. Theoretical Analysis

### 4.1. Stress Mechanism

**Figure 10:**
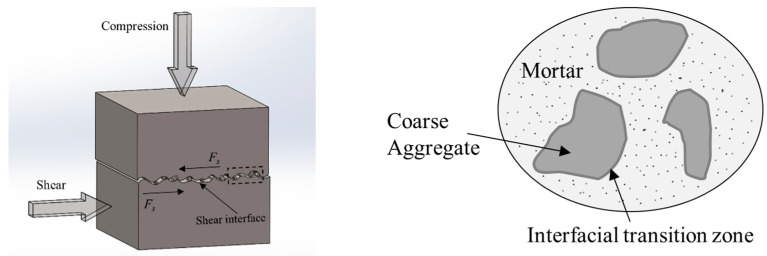
Compression–shear multiaxial stress mechanism model of recycled concrete.

To analyze the compression–shear multiaxial stress mechanism of recycled concrete, the stress model shown in Figure 10 was proposed, according to the experiment plan in this paper and the mechanism of action of RA.

Figure 10 shows the compression–shear multiaxial stress mechanism model of recycled concrete. It can be observed that the shearing action is mainly formed by the van der Waals force, the chemical adhesive force, the mechanical bite force, and the friction force [14]. Since the RA are attached with a certain amount of old cement mortar, the bonding performance between the RA and the mortar is relatively weak. Meanwhile, the cylinder compressive strength of RA is generally lower than that of natural aggregate crushed stone [15]. Thus, the shear stress of recycled concrete is gradually decreased with an increasing RA replacement rate. The axial compressive load has an obvious effect on the mechanical bite force and frictional force. As the axial compressive ratio grows, the mechanical bite force and frictional force on the shear interface will be significantly increased [14]. Von Geel [19] pointed out that the mutual bite force between aggregates was closely related to the distance between adjacent aggregates, while this distance is not only related to the distance between the aggregates before loading, but also related to the distance between the aggregates during the loading process. When RA are mixed into the concrete, affected by the mortar attached to the surface of RA, the deformability of RA is generally higher than that of natural aggregate crushed stone. Under the action of axial compressive loading, recycled concrete is more prone to the evolution of initial defects. Axial compression has a certain weakening effect on the mechanical bite force, and the frictional force on the shear interface. Therefore, as the RA replacement rate increases, the increase in shear stress due to the growth of the axial compression ratio is gradually reduced.

### 4.2. Failure Criterion

#### 4.2.1. Principal Stress Space

The compression–shear multiaxial stress of concrete is composed of the axial compressive stress *σ* and the shear stress *τ*. According to Equations (4) and (5), the axial compressive stress *σ* and the shear stress *τ* can be transformed into the first principal stress *σ*_1_ and the third principal stress *σ*_3_, respectively. Under compression–shear multiaxial action, the second principal stress *σ*_2_ is 0 [14].

(4) $$\sigma_{1}=\frac{\sigma }{2}+\sqrt{{\left(\frac{\sigma }{2}\right)}^{2}+\tau^{2}}$$

(5) $$\sigma_{3}=\frac{\sigma }{2}-\sqrt{{\left(\frac{\sigma }{2}\right)}^{2}+\tau^{2}}$$

**Figure 11:**
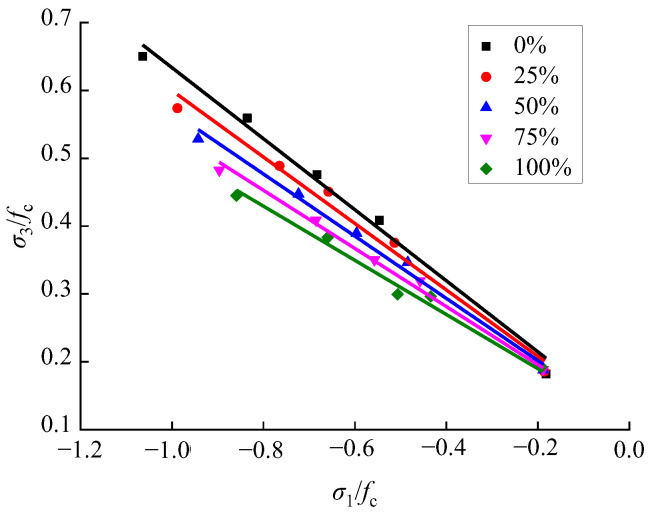
Compression–shear principal stress relationship of recycled concrete.

Based on the compression–shear multiaxial test data of recycled concrete with different RA replacement rates in this article, the first principal stress and the third principal stress were transformed by applying Equations (4) and (5), and the relationship shown in Figure 11 was obtained.

According to Figure 11, the first principal stress and the third principal stress of the recycled concrete basically had a linear relationship under the various RA replacement rates. Thus, the expression shown in Equation (6) was proposed.

(6) $$\frac{\sigma_{3}}{f_{c}}=b+c\times \frac{\sigma_{1}}{f_{c}}$$

**Figure 12:**
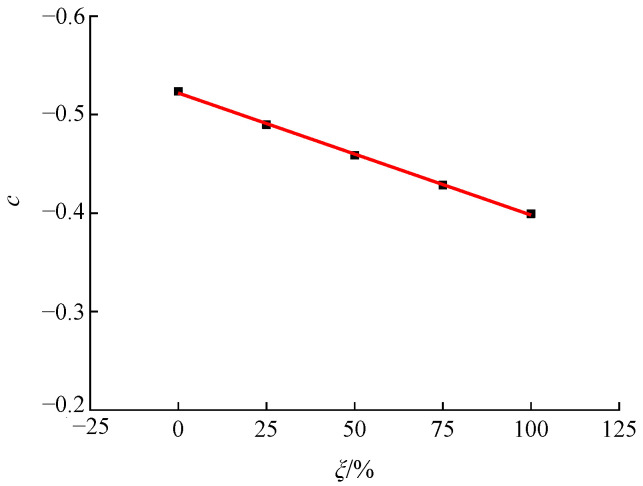
Relationship between the RA replacement rate and parameter c.

Mathematical regression analysis was performed of the relationship between the first and third principal stresses in recycled concrete at different RA replacement rates by applying Equation (6), and the values of parameter b under different replacement rates were obtained, which are basically similar. In order to further simplify Equation (6), the mean value of parameter b corresponding to the five different replacement rates was calculated, to determine its final value, which is 0.11. After substituting the value of 0.11 into Equation (6), a mathematical regression analysis was performed again, by applying the first and third principal stress of recycled concrete under different replacement rates, to determine the value of parameter c, as shown in Figure 12. Since the RA replacement rate *ξ* and the parameter c basically have a linear relationship, the expression shown in Equation (7) was proposed.

(7) $$c=-0.5219+0.0012\times \xi ,\;R^{2}=0.9989$$

By substituting Equation (7) into Equation (6), the relationship between the first and third principal stress of recycled concrete under the influence of the RA replacement rate was obtained, as shown in Figure 11 and Equation (8).

(8) $$\frac{\sigma_{3}}{f_{c}}=0.11-(0.5219-0.0012\times \xi )\times \frac{\sigma_{1}}{f_{c}}$$

#### 4.2.2. Octahedral Stress Space

In the existing literature on the multi-axial mechanical properties of concrete, the proposed failure criteria based on octahedral stress spaces have generally excellent applicability. In the octahedral stress space, the normal stress *σ*_oct_ and the shear stress *τ*_oct_ can be calculated by Equations (9) and (10), respectively [14,16].

(9) $$\sigma_{\mathrm{oct}}=\frac{1}{3}\times (\sigma_{1}+\sigma_{2}+\sigma_{3})$$

(10) $$\tau_{\mathrm{oct}}=\frac{1}{3}\times \sqrt{{\left(\sigma_{1}-\sigma_{2}\right)}^{2}+{\left(\sigma_{2}-\sigma_{3}\right)}^{2}+{\left(\sigma_{3}-\sigma_{1}\right)}^{2}}$$

**Figure 13:**
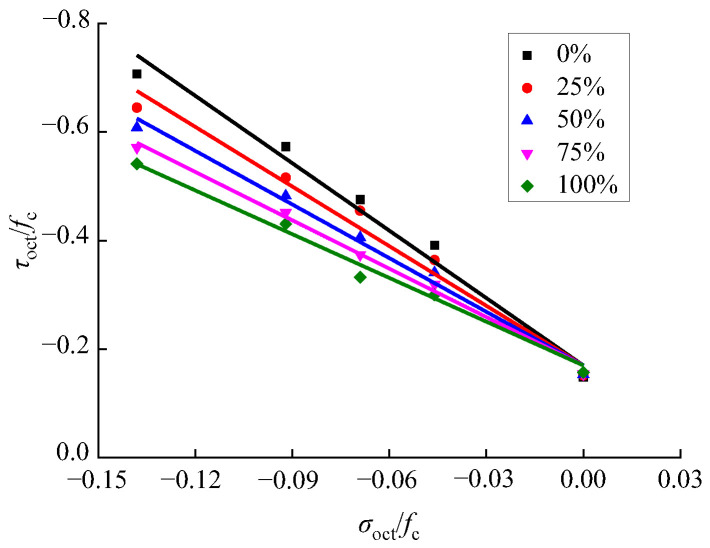
Compression–shear octahedral stress spatial relationship of recycled concrete.

By substituting the first and third principal stress of recycled concrete under different loading conditions into Equations (9) and (10), where the second principal stress is 0, the expression as shown in Figure 13 was obtained.

According to Figure 13, the normal stress and shear stress of recycled concrete basically have a linear relationship under different RA replacement rates. Thus, the expression as shown in Equation (11) was proposed.

(11) $$\frac{\tau_{\mathrm{oct}}}{f_{c}}=d+e\times (\frac{\sigma_{\mathrm{oct}}}{f_{c}})$$

**Figure 14:**
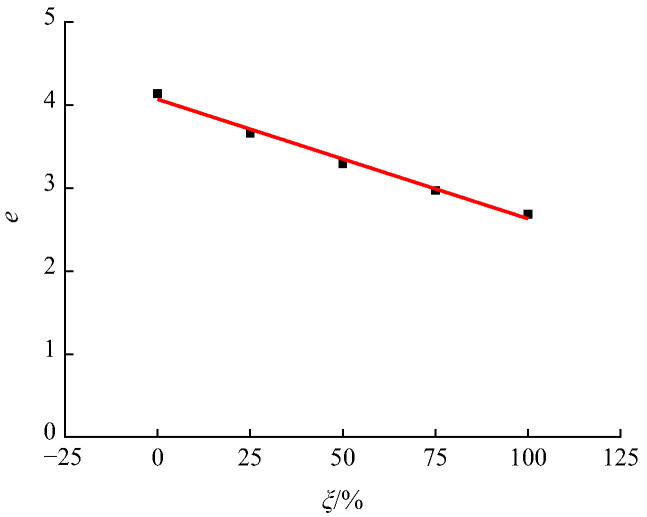
Relationship between the RA replacement rate and parameter *e*.

A mathematical regression analysis of the normal and shear stresses in recycled concrete under different RA replacement rates was carried out by applying Equation (11), and the values of parameter d and e were obtained. Specifically, the values of d are basically similar. In order to further simplify Equation (11), the value of d was set as its mean value, which is −0.17. Then, the normal stress and shear stress data of recycled concrete was used to perform mathematical regression analysis on Equation (11), to determine the value of e under different RA replacement rates, as shown in Figure 14. Since the RA replacement rate *ξ* and parameter *c* basically have a linear relationship, the expression as shown in Equation (12) was proposed.

(12) $$e=4.07-0.0144\times \xi ,\;R^{2}=0.9860$$

By substituting Equation (12) into Equation (11), the relationship between the normal stress and shear stress of recycled concrete under the influence of the RA replacement rate was obtained, as shown in Figure 13 and Equation (13).

By substituting Equation (12) into Equation (11), the relationship between the normal stress and shear stress of recycled concrete under the influence of the replacement rate of RA was obtained, as shown in Figure 13 and Equation (13).

(13) $$\frac{\tau_{\mathrm{oct}}}{f_{c}}=-0.17+(4.07-0.0144\times \xi )\times (\frac{\sigma_{\mathrm{oct}}}{f_{c}})$$

**Figure 15:**
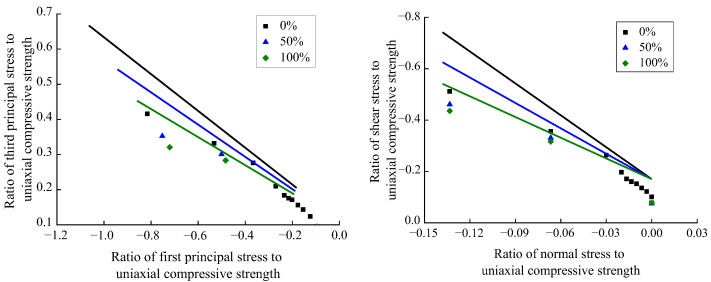
Comparison between the failure criterion proposed in this paper and the reference data.

Yu et al. [14] investigated the compressive–shear multiaxial mechanical properties of ordinary concrete. Wang et al. [16] examined the compressive–shear multiaxial mechanical properties of recycled concrete at the RA replacement rates of 0%, 50%, and 100%. The compression–shear multiaxial test data in the above-mentioned studies were preprocessed and then compared with the failure criterion proposed in this article, as shown in Figure 15.

According to Figure 15, based on the compressive stress and shear stress data obtained from the compression–shear multiaxial test in reference [14], the developing trends of the relationships between the first and third principal stresses and between the normal stress and shear stress are consistent with the failure criterion proposed in this paper. In reference [14], the compression–shear multiaxial test data of ordinary concrete is in good agreement with the failure criterion under the condition of 0% RA replacement rate in this paper. In reference [16], the compression–shear test data of recycled concrete at the RA replacement rates of 0%, 50%, and 100% deviate from the failure criterion proposed in this paper to a certain extent. The mechanism for such deviations is mainly attributed to the differences in the physical properties of RA and the influence of the bending effect, rotation effect, and friction effect on the accuracy of trial data. Due to the combined factors above, Guo [20] pointed out that the shear stress data obtained by different test techniques may differ by two to three times.

### 4.3. Calculation Mode

Hai-feng, Y et al. [21] pointed out that the shear stress of ordinary concrete was mainly composed of the cohesive shear stress and the interfacial shear stress, and the calculation model shown in Equation (14) was established.

(14) $$\tau_{\mathrm{pk},0}=f_{\mathrm{cu}}\times (\alpha_{1}\sqrt{\alpha_{2}+\alpha_{3}k+\alpha_{4}k^{2}}+\alpha_{5}k+\alpha_{6})$$

where *τ*_pk,0_ is the shear stress of ordinary concrete; *f*_cu_ is the concrete compressive strength; *k* is the concrete axial compression ratio; and *α*_1_–*α*_6_ are undetermined coefficients.

From the analysis above, it can be seen that the shear stress of recycled concrete is mainly affected by the axial compression ratio *σ/f*_c_ and the RA replacement rate *ξ*. Thus, according to Equation (14), a shear stress calculation model for recycled concrete under the influence of RA replacement rate and axial compression ratio was proposed by taking into account the influencing factors of axial compression ratio *λ* and RA replacement rate *μ*. In this paper, *λ* has a linear relationship with the axial compression ratio and *μ* has a linear relationship with the RA replacement rate. Thus, the expressions shown in Equations (15) and (16) were proposed.

(15) $$\lambda =a_{1}\times k+1$$

(16) $$\mu =a_{2}\times \xi +1$$

By multiplying Equations (15) and (16) with Equation (14), the formula for calculating the shear stress of recycled concrete was obtained, as shown in Equation (17).

(17) $$\tau_{\mathrm{pk}}=f_{\mathrm{cu}}\times (\alpha_{1}\sqrt{\alpha_{2}+\alpha_{3}k+\alpha_{4}k^{2}}+\alpha_{5}k+\alpha_{6})\times (a_{1}\times k+1)\times (a_{2}\times \xi +1)$$

where *τ*_pk_ is the shear stress of recycled concrete.

By applying the test data in this paper to perform a mathematical regression analysis on Equation (17), the calculation equation for the shear stress of recycled concrete under the influence of axial compression ratio and RA replacement rate was obtained, as shown in Equation (18).

(18) $$\begin{array}{l} \tau_{\mathrm{pk}}=f_{\mathrm{cu}}\times (-1.89\times \sqrt{0.91-5.94k+10.39k^{2}}-8.71k+1.59)\\ \times (1-1.74\times k)\times (1-0.27\times \xi ) \end{array}$$

**Figure 16:**
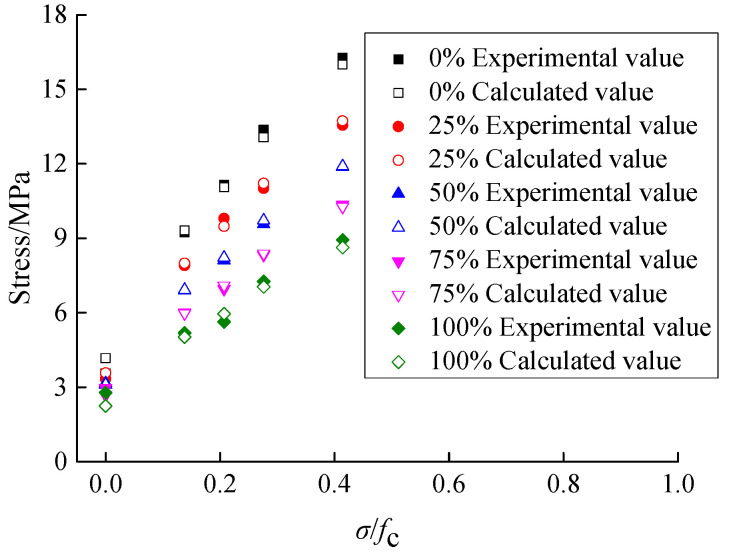
Comparison between the test data and the calculated values of shear stress of recycled concrete.

According to the parameters of axial compression ratio and RA replacement rate, and the uniaxial compressive strength data in this paper, Equation (18) was applied to calculate the theoretical shear stress values of recycled concrete, which were compared with the test data, as shown in Figure 16.

Figure 16 shows a comparison between the test data and the calculated values of shear stress of recycled concrete. It can be seen that the theoretical values obtained from Equation (18) are basically similar to the test data, with an average deviation of 3.67%. Thus, the proposed model equation has good applicability for calculating the shear stress of recycled concrete.

## 5. Conclusions

As an eco-friendly building material, recycled concrete is often subjected to a compression–shear multiaxial stress state in practical structural applications. In view of such fact, a systematic study was carried out on the compression–shear multiaxial mechanical properties of recycled concrete in this paper, by considering the influencing factors of RA replacement rate and axial compression ratio. Eventually, the following conclusions were reached:

(1) According to analysis of the macroscopic failure modes, it was found that the shear cracks of test specimens with different replacement rates all exhibit a developing trend along the oblique direction with the growth of axial compression ratio, and the friction traces on the shear interface are gradually deepened. As the RA replacement rate increases, the number and size of cracks tend to increase gradually.
(2) From the perspective of shear stress and shear strain, with the growth of axial compression ratio, the shear stress and shear strain of recycled concrete are gradually increased. With the increase of the RA replacement rate, the shear stress is gradually reduced, while the shear strain is gradually increased. In addition, the variation range of shear stress and shear strain under the influence of axial compression ratio are also gradually reduced.
(3) According to the shear stress test data under different RA replacement rates and axial compression ratios, a compression–shear multiaxial failure criterion under the influence of RA replacement rate and a shear stress calculation model for recycled concrete were proposed. Both the failure criterion and the calculation model have good applicability to engineering practice.

## Figures and Tables

**Table 1:** Mix ratios of recycled concrete (unit: kg/m^3^).

Replacement Rate	Water	Cement	Fine Aggregates	Coarse Aggregates	
				NCA	NCA
0%	175	461	512	1252	0
25%	175	461	512	939	313
50%	175	461	512	626	626
75%	175	461	512	313	939
100%	175	461	512	0	1252

## Data Availability

Not applicable.
